# Bacteria and Genes Involved in Arsenic Speciation in Sediment Impacted by Long-Term Gold Mining

**DOI:** 10.1371/journal.pone.0095655

**Published:** 2014-04-22

**Authors:** Patrícia S. Costa, Larissa L. S. Scholte, Mariana P. Reis, Anderson V. Chaves, Pollyanna L. Oliveira, Luiza B. Itabayana, Maria Luiza S. Suhadolnik, Francisco A. R. Barbosa, Edmar Chartone-Souza, Andréa M. A. Nascimento

**Affiliations:** 1 Departamento de Biologia Geral, Instituto de Ciências Biológicas, Universidade Federal de Minas Gerais; Belo Horizonte, Brazil; 2 Grupo de Genômica e Biologia Computacional, Centro de Pesquisas René Rachou (CPqRR), Fundação Oswaldo Cruz (FIOCRUZ), Belo Horizonte, Brazil; Université Claude Bernard - Lyon 1, France

## Abstract

The bacterial community and genes involved in geobiocycling of arsenic (As) from sediment impacted by long-term gold mining were characterized through culture-based analysis of As-transforming bacteria and metagenomic studies of the *arsC*, *arrA*, and *aioA* genes. Sediment was collected from the historically gold mining impacted Mina stream, located in one of the world’s largest mining regions known as the “Iron Quadrangle”. A total of 123 As-resistant bacteria were recovered from the enrichment cultures, which were phenotypically and genotypically characterized for As-transformation. A diverse As-resistant bacteria community was found through phylogenetic analyses of the 16S rRNA gene. Bacterial isolates were affiliated with *Proteobacteria*, *Firmicutes*, and *Actinobacteria* and were represented by 20 genera. Most were AsV-reducing (72%), whereas AsIII-oxidizing accounted for 20%. Bacteria harboring the *arsC* gene predominated (85%), followed by *aioA* (20%) and *arrA* (7%). Additionally, we identified two novel As-transforming genera, *Thermomonas* and *Pannonibacter*. Metagenomic analysis of *arsC*, *aioA*, and *arrA* sequences confirmed the presence of these genes, with *arrA* sequences being more closely related to uncultured organisms. Evolutionary analyses revealed high genetic similarity between some *arsC* and *aioA* sequences obtained from isolates and clone libraries, suggesting that those isolates may represent environmentally important bacteria acting in As speciation. In addition, our findings show that the diversity of *arrA* genes is wider than earlier described, once none *arrA-*OTUs were affiliated with known reference strains. Therefore, the molecular diversity of *arrA* genes is far from being fully explored deserving further attention.

## Introduction

Arsenic occurs naturally in the earth’s crust and is widely distributed in the environment [1], [2]. Natural mineralization and microorganisms enhance arsenic mobilization in the environment, but human interventions, such as gold mining, have aggravated the environmental arsenic contamination arousing health concerns. Water pollution by arsenic is one of the major challenges for public health, primarily due to its carcinogenic potential at low doses [3], [4], [5], [6]. According to Nordstrom [7] over 50 million people in the world are at risk from drinking arsenic-contaminated water. Moreover, given that arsenic has a variety of valence states (+V, +III, 0, −III) with different physicochemical properties, the removal of arsenic from contaminated water bodies is yet a challenge.

In nature, microorganisms have developed different response mechanisms to metabolize As, mainly via reduction and oxidation reactions, leading to its speciation [8]. Previous studies have regarded As speciation as a result of microbial activity in the environment, including some derived from gold-mining activities [1], [2]. However, few bacterial genera involved in As transformation have been found at any of the sites studied [9]–[13]. Thus, a more comprehensive knowledge on the structure of the bacterial community involved in As-transformation in gold-mining sites remains warranted.

The arsenate (AsV) reducing pathways known are the detoxification (*arsC* gene) and the dissimilatory respiration (*arrA/B* genes). The organization of *ars* operons varies greatly between taxa, and the core genes include *arsR*, *arsB* and *arsC*, whereas *arsD* and *arsA* genes can eventually be found [1]. The *ars*C gene encodes the enzyme AsV reductase, which is located in the cytoplasm and is responsible for the biotransformation of AsV to AsIII. This enzyme together with a transmembrane efflux pump, encoded by *arsA* and *arsB* genes, is the most common As transformation mechanism in the environment [2], [14]–[16]. Moreover, *arrA*/*B* genes encode a periplasmic AsV reductase that works during anaerobic respiration using AsV as the final electron acceptor for energy generation [17]. The AsV dissimilatory respiration reduction has already been described for many bacterial phyla, including obligatory and facultative anaerobic bacteria and some archaea [1].

The microbial oxidation of AsIII was first reported in 1918 and can be mediated by two distinct enzymes: AioBA, hardly studied, and ArxAB, recently described by Zargar et al. [18]. Both enzymes have been found in several heterotrophic and chemolithoautotrophic bacterial species [19]–[22]. Aerobic AsIII oxidation is catalyzed by arsenite oxidase, which uses O_2_ as terminal electron acceptor, and is encoded by *aioB/A* genes, formerly referred to as *aoxA/B*, *aroB/A* and *asoB/A* genes [20], [23]. ArxAB detected in AsIII oxidizing bacteria in anoxic conditions, in which nitrate or chlorate reduction is coupled to AsIII oxidation in the chemolithotrophs [24], [25]. Interestingly, members of the genus *Ectothiorhodospira* are able to use AsIII as electron donor for anoxygenic phototrophic growth [26]. According to Zargar et al. [18] the *arxA* gene is more closely related to *arrA* than to *aioA* genes.

In this research, we bioprospected As-resistant bacteria from As-enrichment culture of sediments collected from a stream located at the Brazilian gold mining area known as the Iron Quadrangle (IQ, Minas Gerais state), one of the world’s largest mining regions. Much concern exists about As-contamination of gold-mining sites in this area because it is estimated that at least 390,000 tons of As have been released into this area since the beginning of gold-mining activity in the 17th century [27]. We also investigated the diversity of As-transforming genes using metagenomic strategies. This included the genes for arsenite oxidase (*aioA*) and arsenate reductases (*arsC* and *arrA*).

## Materials and Methods

### Ethics Statement

For sampling in Mina stream, no specific permit was required for the described field study. The study location is not privately-owned or protected in any way and we confirm that the field study did not involve endangered or protected species.

### Study Area and Sampling

Mina stream (19°58′46.80″S–43°49′17.07″W) is a natural body of water located at the Velhas River Basin (IQ, Minas Gerais state, Brazil) and characterized as backwater (Figure S1). This stream was chosen because is located near a historically impacted gold-mining area. Moreover, previous investigations [28] reported As concentrations superior to those permitted by Brazilian law (Conselho Nacional do Meio Ambiente – CONAMA) and by Canadian Environmental Quality Guidelines (Canadian Council of Ministers of the Environment– CCME).

Bulk water and superficial sediment samples (up to 1.0 cm depth) were collected on 13 July 2011, during the dry season. The typical sediment core can be divided into three zones: oxic, suboxic and anoxic [29]. According to literature the thick oxic zone can extend from several mm up to 10 cm [30], [31]. In this work the sampling site was shallow (20 cm) and therefore highly influenced by the nutrients and oxygen concentrations of the water body. The analyzed sediment was taken from the upper part, representing the oxic zone. Samples were collected aseptically at three points at 1m distance from each other, subsequently pooled in a single sample, and stored at 4°C for bacterial analysis or at −20°C for chemical and molecular analyses.

To assess the bulk water conditions physicochemical characteristics such as temperature, pH, and dissolved oxygen (DO) concentration were measured *in situ* with a multiprobe (Horiba, model U-22) [30]. Concentrations of total nitrogen (TN), total phosphorus (TP), ammonium (NH_4_^+^-N), nitrite (NO_2_-N), nitrate (NO_3_-N), and soluble reactive phosphorus (PO_4_-P) were measured as previously described [32], [33]. Metal and metalloid concentrations of water and sediment samples were determined by using an inductively coupled plasma-optical emission spectrometer (ICP-OES, Optima 7300 DV, PerkinElmer).

### Arsenic Enrichment and Isolation

Sediment (10 g) samples were added to Erlenmeyer flasks containing 100 mL of CDM medium (0.012 mM Fe_2_SO_4_, 7 mM Na_2_SO_4_, 0.0574 mM K_2_HPO_4_, 9.5 mM NaHCO_3_, 18.7 mM NH_4_Cl, 8.12 mM MgSO_4_, 0.457 mM CaCl_2_ and 44.6 mM sodium lactate as organic carbon source, pH 7.2) with either 2 mM sodium arsenite or 10 mM sodium arsenate and incubated at 28°C for seven days. Then, serial 10-fold dilutions of the enrichment cultures were plated onto CDM agar media (1.5% agar) amended with 2 mM sodium arsenite or 10 mM sodium arsenate to selectively enrich and isolate AsIII- and AsV-resistant bacteria. Plates were incubated at 28°C for five days. The resulting colonies were repeatedly streaked on the same medium to accomplish their purification. The bacterial isolates from AsIII- and AsV-resistant bacteria (named MS-AsIII and MS-AsV, respectively) were stored at −20°C in 25% glycerol.

### DNA Extraction from the Cultures and Sediment

Genomic DNA was extracted and purified from each MS-AsIII and MS-AsV isolate using a protocol previously described [34]. Additionally, metagenomic DNA was extracted from 10 g (wet weight) of sediment using the PowerSoil DNA Extraction Kit (MO BIO Laboratories, USA) according to the manufacturer’s instructions. Total DNA from the MS-AsIII and MS-AsV isolates and sediment were quantified by absorbance at 260 nm using a NanoDrop Spectrophotometer (NanoDrop Technologies). DNA purity was assessed using the A260/A280 and A260/A230 ratios. DNA was stored at −20°C until further processing.

### PCR Amplification and Construction of Clone Libraries

Briefly, touchdown PCR was carried out by amplifying bacterial MS-AsIII and MS-AsV isolates 16S rRNA gene fragments using the conditions previously described by Freitas *et al*. [35]. The reactions were performed using the bacterial-targeted primer set 8F (5′-AGAGTTTGATYMTGGCTCAG-3′) and 907R (5′-CCGTCAATTCMTTTRAGT-3′) [36]. Taq DNA polymerase and dNTPs were purchased from Fermentas (Canada) and used in all the PCR reactions.

Metagenomic and genomic DNA were used as template for PCR employing the *arsC*, *arrA* and *aioA* genes for construction of clone libraries and genotypic characterization of the bacterial MS-AsIII and MS-AsV isolates. PCR reactions targeting the *arsC*, *arrA* and *aioA* genes were carried out using primers and conditions as previously described by Sun *et al.* [37], Malasarn *et al.* [17] and Hamamura *et al.* [20], respectively. The *arsC* gene examined was of the glutaredoxin-dependent arsenate reductase enzyme, ArsC, from *Escherichia coli* R773 plasmid. The primer chosen has been successfully applied in several investigations of a variety of environmental samples [37], [38].

The amplicons of *arsC*, *arrA*, and *aioA* genes were gel-purified using the Silica Bead DNA Gel Extraction Kit (Fermentas, Canada). PCR products were cloned into the vector pJET1.2/blunt (Fermentas, Canada), and propagated with *Escherichia coli* XL1-Blue electrocompetent cells according to the manufacturer’s instructions.

### Sequencing and Phylogenetic Analysis

Partial 16S rRNA, *arsC*, *arrA*, and *aioA* gene sequences were obtained using BigDye Terminator Cycle Sequencing kit (Life Technologies, USA) according to the manufacturer’s instructions. The nucleotide sequences were quality checked and submitted to GenBank with accession numbers from KC577613 to KC577798. The 16S rRNA gene sequences were analyzed through blastn (http://www.ncbi.nlm.nih.gov) and Classifier search tool (http://rdp.cme.msu.edu) to determine their phylogenetic affiliation. The *arsC*, *arrA*, and *aioA* gene sequences were compared with those available at the GenBank databases using blastn and blastx tools (http://www.ncbi.nlm.nih.gov) to retrieve potential homologs. Operational taxonomic unities (OTUs) from As gene clone libraries were defined with DOTUR software [39] using a cut-off threshold of ≥97% identity. Coverage of the clone libraries was calculated using the equation C = 1−(n/N)×100, where n is the number of unique OTUs and N is the number of sequences analyzed in the library [40].

In total, five fasta files were obtained containing *arsC*, *arrA*, *aioA*, MS-AsIII 16S rRNA, and Ms-AsV 16S rRNA gene sequences. Due to the short length of *arsC* and *arrA*amino acid sequences obtained in this study, added to the high similarity of some OTUs and isolates, we decided to reconstruct the phylogenetic relationships of As metabolism genes using nucleotide sequences to increase the phylogenetic signal and avoid overparameterization.

Sets of nucleotide sequences were independently aligned using MAFFT 7 with iterative refinement by the G-INS-i strategy [41]. Multiple sequence alignments were manually refined using Jalview [42]. To optimize the datasets for evolutionary analyses we removed redundancy and sequences too distantly related using the Decrease Redundancy tool available as a resource at ExPaSy (www.expasy.org). The Decrease Redundancy parameters were set as 99 for “% max similarity” and 30 for “% min similarity”. Identical sequences were clustered as single OTUs and filtered alignments were further used in phylogenetic analyses. Identifiers of filtered sequences were later included into the phylogenetic tree. To reconstruct phylogenetic trees we used the maximum likelihood method (ML) as implemented in PhyML [43]. For the phylogenetic reconstruction we tested seven different evolutionary models (HKY85, JC69, K80, F81, F84, TN93, and GTR) using the jModelTest 2 software [44]. The evolutionary model best fitting the data was determined by comparing the likelihood of tested models according to the Akaike Information Criterion (AIC). Statistical support value for each node was computed by approximate likelihood ratio test (aLTR). Trees were visualized and edited using the FigTree software (tree.bio.ed.ac.uk/software/figtree).

### Susceptibility and Arsenic Transformation Tests

Minimum inhibitory concentrations (MIC) were established, in triplicate, by the agar dilution method in CDM with 1×10^5^ CFU ml^−1^ as standard inoculums. CDM plates were supplemented with increasing concentrations (from 2 mM to 1024 mM) of AsIII or AsV and incubated at 28°C for seven days. MIC was defined as the lowest AsIII or AsV concentration that completely inhibited bacterial growth.

The ability to oxidize AsIII and reduce AsV was investigated using a qualitative screening according to [45]. To achieve that, bacterial MS-AsIII and MS-AsV isolates were grown in CDM broth with 100 mg l^−1^ 2 mM sodium arsenite or 100 mg l^−1^ sodium arsenate until an optical density of 0.4 at 595 nm was reached. After that, 20 µl of 0.01 mol l^−1^ of potassium permanganate solution were added in 1 ml of bacterial culture. The data were interpreted according to the change in medium color, i.e., a pink color indicated a positive oxidation of AsIII and a yellow color indicated a positive reduction of AsV.

## Results

### Environmental Parameters

The physicochemical characteristics of the water and sediment samples from the Mina stream are presented in Tables 1 and 2. Data displayed on Table 1 revealed that metal concentrations in the Mina stream exceeded the maximum allowable concentrations established by Brazilian and Canadian environmental regulations [46], [47] for sediment and water. Al, Mn, Fe, Cu, As and Zn were the metals present in the highest concentrations in the sediment sample analyzed.

**Table 1:** Metal concentration from sediment and water of Mina Stream and limits permitted by law.

Metals	Sediment (mg kg^−1^)	CONAMA* (mg kg^−1^)	Water (mg l^−1^)	CONAMA** (mg l^−1^)
Fe	492.8	NE	0.52	15
Ni	9.0	18	<0.1	2
Mn	1284.5	NE	1.45	1
Cu	387.7	35.7	0.19	1
Pb	8.7	35	NE	0.5
Cd	<2.5	0.6	<0.1	0.2
Zn	180.9	123	0.2	5
Al	2343.2	NE	<0.5	NE
As	297.1	5.9	<0.1	0.5
Cr	17.3	37.3	<0.1	1
Hg	<2.5	0.17	<0.1	0.01

NE – Not established.

*CONAMA resolution 344/04.

**CONAMA resolution 430/11.

**Table 2:** Physicochemical parameters from water of Mina Stream.

Parameters	Water
pH	6.2
Conductivity (µs cm^−1^)	2151
Temperature (°C)	18.0
Dissolved Oxygen (mg l^−1^)	9.1
Redox (mV)	215
NO_3_^−^ -N (µg l^−1^)	3103.8
NO_2_^−^ -N (µg l^−1^)	161.3
NH_4_^+^ -N (µg l^−1^)	829.5
PO_4_^3−^ -P (µg l^−1^)	2.3
Total P (µg l^−1^)	77.6
Total N (µg l^−1^)	2916.8

The physicochemical analysis revealed that the Mina stream can be characterized as a mesothermal oxidized environment with highly oxygenated and circum-neutral waters (Table 2). Nitrogen and phosphorus ratio was greater than nine (Table 2). According to Salas & Martino [48], this ratio indicates that the phosphorus was the most limiting nutrient and that the stream can be classified as eutrophic.

### Phylogenetic Affiliation

**Figure 1:**
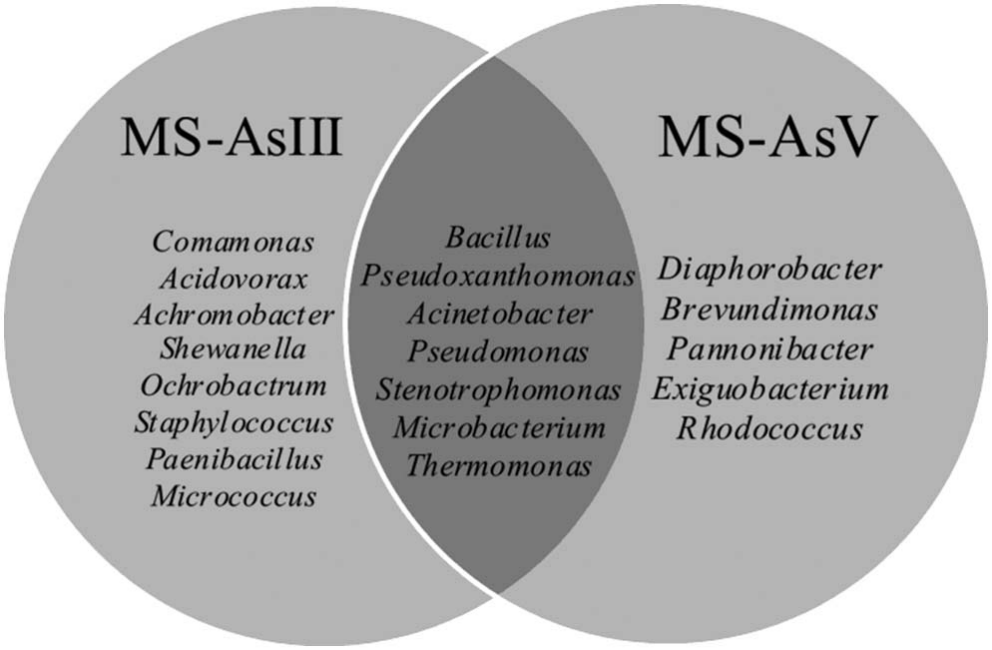
Venn diagram showing the exclusive and shared bacterial genera retrieved from MS-AsIII and MS-AsV enrichment cultures.

In total, 123 bacterial isolates were recovered from the enrichment cultures (68 and 55 from the MS-AsIII and MS-AsV, respectively). Partial 16S rRNA gene sequences used for phylogenetic analysis were approximately 600 bp long and spanned the V2 to V4 variable regions. MS-AsIII and MS-AsV isolates were categorized into three phyla: *Proteobacteria* (56% and 59%, respectively, includes *alpha*, *beta*, and *gamma-Proteobacteria*), *Firmicutes* (36% in both enrichment cultures), and *Actinobacteria* (8% and 5%). Twenty genera represented these phyla in the Mina stream sample analyzed. Differences in the bacterial composition between the MS-AsIII and MS-AsV enrichment cultures were detected (Table 3 and Figure 1). The resulting Venn diagram shows that a higher bacterial diversity was observed in the MS-AsIII than in the MS-AsV enrichment cultures. Eight genera were specifically found in MS-AsIII and seven were shared between the culture systems (Figure 1).

**Table 3:** Phylogenetic distribution of the bacterial isolates and their As-metabolism phenotype and genotype.

Enriched culture	Phylum	Genus	N° of isolates*	Phenotype**	Genotype***
MS-AsV	*Proteobacteria*	*Acinetobacter*	1	reducer	*arsC aioA*
		*Brevundimonas*	9	reducer (3)	*arsC* [9] *aioA* [1]
		*Diaphorobacter*	1	reducer	*arsC*
		*Pannonibacter*	2	reducer (2)	*arsC* [2]
		*Pseudomonas*	6	reducer (2)	*arsC* [6] *aioA* [2]
		*Pseudoxanthomonas*	10	reducer (6)	*arsC* [7] *aioA* [1]
		*Stenotrophomonas*	5	reducer (5)	*arsC* [5] *aioA* [2]
		*Thermomonas*	1	reducer	*arsC*
	*Firmicutes*	*Bacillus*	14	reducer (9), oxidizer (8)	*arsC* [12] *aioA* [6] *arrA* [1]
		*Exiguobacterium*	1	reducer	*arsC aioA*
	*Actinobacteria*	*Microbacterium*	4	reducer (4) oxidizer (1)	*arsC* [3] *aioA* [1]
		*Rhodococcus*	1	reducer	*arsC*
MS-AsIII	*Proteobacteria*	*Achromobacter*	1	oxidizer	*arsC aioA*
		*Acidovorax*	2	oxidizer(2)	*arsC* [2] *aioA* [1]
		*Acinetobacter*	4	reducer (3)	*arsC* [2] *aioA* [1]
		*Comamonas*	5	reducer (5)	*arsC* [5] *arrA* [2]
		*Ochrobactrum*	1	reducer	*arsC*
		*Pseudomonas*	3	reducer (3)	*arsC* [1] *aioA* [1]
		*Pseudoxanthomonas*	17	reducer (9) oxidizer (5)	*arsC* [14] *aioA* [2] *arrA* [1]
		*Shewanella*	1	reducer	*arrA*
		*Stenotrophomonas*	2	reducer (2) oxidizer (1)	*arsC* [1] *aioA* [1]
		*Thermomonas*	2	reducer (1)	*arsC* [2] *arrA* [1]
	*Firmicutes*	*Bacillus*	21	reducer (19) oxidizer (5)	*arsC* [21] *aioA* [2] *arrA* [3]
		*Paenibacillus*	1	reducer oxidizer (1)	*arsC*
		*Staphylococcus*	4	reducer (4) oxidizer (1)	*arsC* [3]
	*Actinobacteria*	*Micrococcus*	3	reducer (2) oxidizer (1)	*arsC* [1] *aioA* [1]
		*Microbacterium*	1	reducer	*-*

*The number represents the total of bacterial isolates identified.

**Values in parentheses indicate the number of As-redox isolates.

***Values in bracket indicate the number of isolates harboring As-metabolism genes.

Dominant genera in MS-AsV were *Bacillus* (26%), *Pseudoxanthomonas* (18%), and *Brevundimonas* (16%). The predominant population in MS-AsIII was *Bacillus* (30%), followed by *Pseudoxanthomonas* (25%). The other bacteria related to MS-AsIII and MS-AsV are listed in Table 3. Although the *Proteobacteria* phylum was the most diverse and dominant, the *Bacillus* (29%) genus was the most abundant and diverse among the genera because it harbored eight identified species.

### Characterization of As-reducing and Oxidizing Isolates and Identification of their Genes Involved in As Metabolism

The MICs for the MS-AsIII and MS-AsV isolates were determined. The highest MIC was found for AsV in which 94% of the isolates exhibited values ≥256 mM, whereas 90% of the isolates displayed MICs ranging from 32 mM to 64 mM for the most toxic AsIII.

The As-transformation ability of the isolates was determined with a qualitative test that revealed that 72% of the isolates were AsV-reducing, whereas 20% were AsIII-oxidizing. Of those, 8% presented AsV-reducing as well as AsIII-oxidizing activities. Among the 20 genera identified in both MS-AsIII and MS-AsV enrichment cultures, *Acidovorax* and *Achromobacter* presented only AsIII-oxidizing activity. No As-transformation activity was found in 8% of the total of MS-AsIII and MS-AsV isolates (123) (Table 3).

The molecular analysis of the MS-AsIII and MS-AsV isolates unveiled that the *arsC* gene was the most frequent (85%), followed by *aioA* (20%) and *arrA* (7%) (Table 3). Of those, *Bacillu*s was the only genus harboring all three genes, and *Shewanella* was the only genus which did not harbor the most common gene (*arsC*) in the isolate analyzed (MS-AsIII-61). *Achromobacter* and *Acidovorax* both harbored the *aioA* gene, confirming the phenotypic data. *Thermomonas* and *Pannonibacter* also harbored As resistance genes.

### General Features of Clone Libraries

To unveil the molecular diversity of genes involved in As metabolism in the Mina stream sediment, three clone libraries for *arsC*, *arrA*, and *aioA* genes were constructed. One hundred sixty-four sequences were analyzed after quality control and the removal of chimeric sequences. The coverage values of the three libraries (80%, 70% and 63%, respectively for *arsC*, *arrA*, and *aioA*) indicated that most of the diversity of these genes was detected.

Blastx analysis of *arsC, aioA*, and *arrA*-OTUs revealed high similarity with sequences from glutaredoxin-glutathione arsenate reductase (from 76 to 100%), molybdopterin-binding arsenite oxidase (from 71 to 96%), and respiratory arsenate reductase (from 64 to 98%) (Tables S1, S2, and S3 in Tables S1). The sequences corresponding to *arsC* were associated with *arsC* harboring different bacterial taxa from a variety of environments. The *aioA*-OTUs were closely related to uncultured and cultured clones from As contaminated environments. Furthermore, all *arrA*-OTUs were closely related to uncultured clones from rock biofilms of an ancient gold mine and Cache Valley Land Fill sediments, both arsenic-contaminated environments.

### Phylogenetic Analyses of 16S rRNA, *arsC*, *aioA*, and *arrA* Genes Sequences

**Figure 2:**
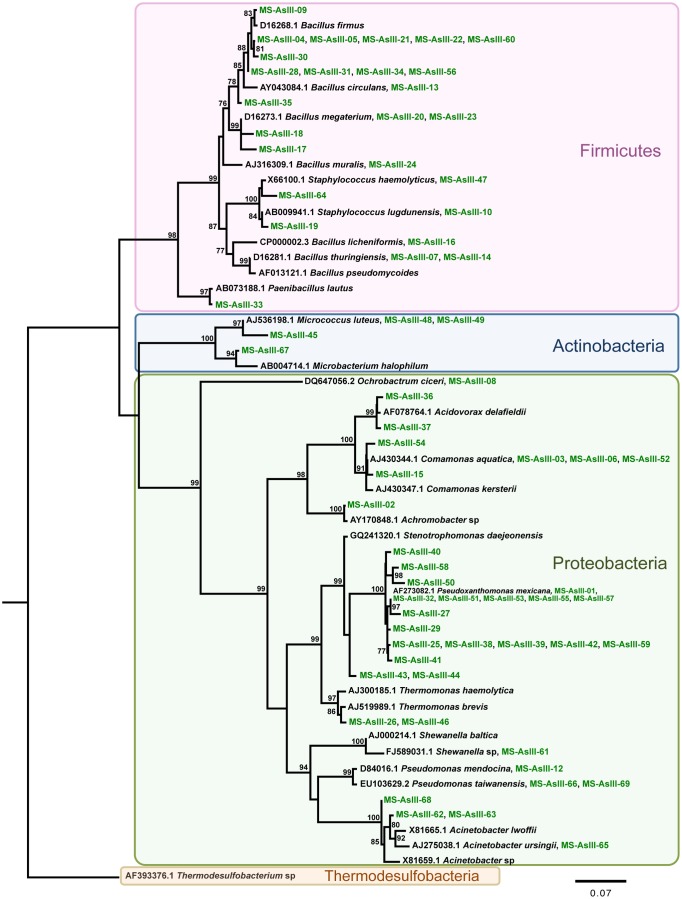
Evolutionary relationships of AsIII-resistant bacteria (MS-AsIII) 16S rRNA sequences. A total of 57 nucleotide sequences and 719 sites were analyzed. The phylogeny was reconstructed by maximum likelihoodand TIM3+I+G+F was selected as best fit model. Support values for each node were estimated using the Akaike Likelihood Ratio Test (aLRT). Only support values higher than 70% are shown. Reference sequences retrieved from the non-redundant database of the NCBI are shown in black, bacterial isolates (MS-AsIII and MS-AsV) in green. Different background colors highlight three well-supported clades: *Firmicutes*, *Actinobacteria*, and *Proteobacteria*. *Thermodesulfobacteria* was used as outgroup.

**Figure 3:**
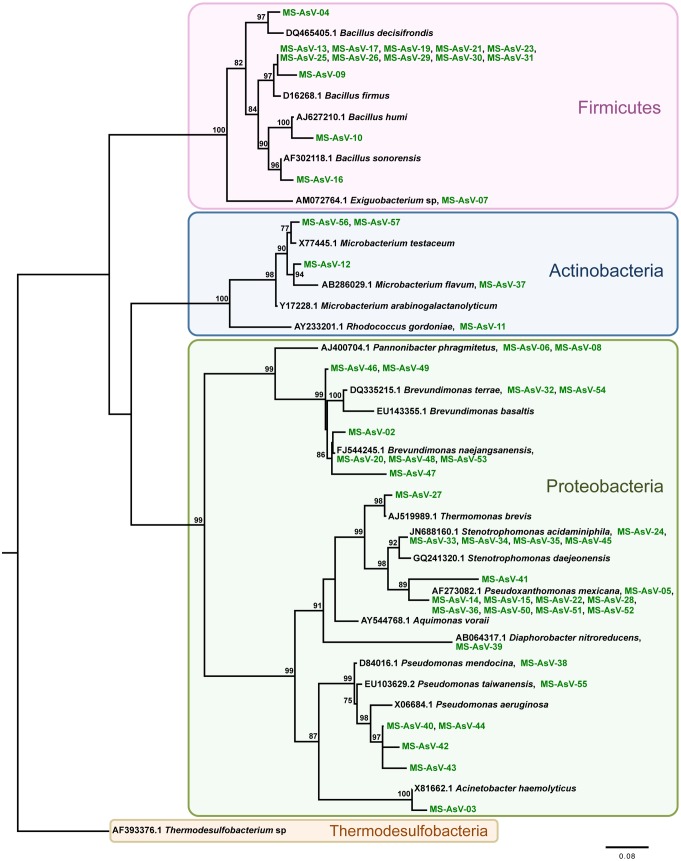
Evolutionary relationships of AsV-resistant bacteria (MS-AsV) 16S rRNA sequences. A total of 40 nucleotide sequences and 721 sites were analyzed. The phylogeny was reconstructed by maximum likelihood and TrN+G+F was selected as best fit model. Support values for each node were estimated using the Akaike Likelihood Ratio Test (aLRT). Only support values higher than 70% are shown. Reference sequences retrieved from the non-redundant database of the NCBI are shown in black, bacterial isolates (MS-AsIII and MS-AsV) in green. Different background colors highlight three well-supported clades: *Firmicutes*, *Actinobacteria*, and *Proteobacteria*. *Thermodesulfobacteria* was used as outgroup.

In this study we have amplified, sequenced and reconstructed the evolutionary relationships of 16S rRNA and As metabolism genes encoded by As-resistant bacteria retrieved from a stream located at the Brazilian gold mining area and cultivated on As-enrichment sediment’s culture, as well as As metabolism genes of clone libraries. The phylogeny of the AsIII-resistant bacteria (MS-AsIII) 16S rRNA gene sequences was reconstructed from an alignment containing 57 operational taxonomic units and 719 sites, which represent 99 sequences (Fig. 2). Therefore, 42 sequences were considered redundant by the Decrease Redundancy tool (www.expasy.org). The phylogenetic tree reconstructed by using the maximum likelihood method as implemented in PhyML [43], shows sequence’s clear separation into three strongly supported clades, which have representatives of the *Firmicutes*, *Actinobacteria*, and *Proteobacteria* phyla (Fig. 2). Similar results were obtained for the AsV-resistant bacteria (MS-AsV) 16S rRNA phylogenetic analysis (Fig. 3). The evolutionary history was based on an alignment containing 40 OTUs and 721 sites, representing 79 sequences (Fig. 3). The Decrease Redundancy tool filtered about 50% of the initially selected sequences. The resulting phylogeny also exhibits the presence of three well-supported clades containing *Firmicutes*, *Actinobacteria*, and *Proteobacteria* phyla representatives (Fig. 3).

**Figure 4:**
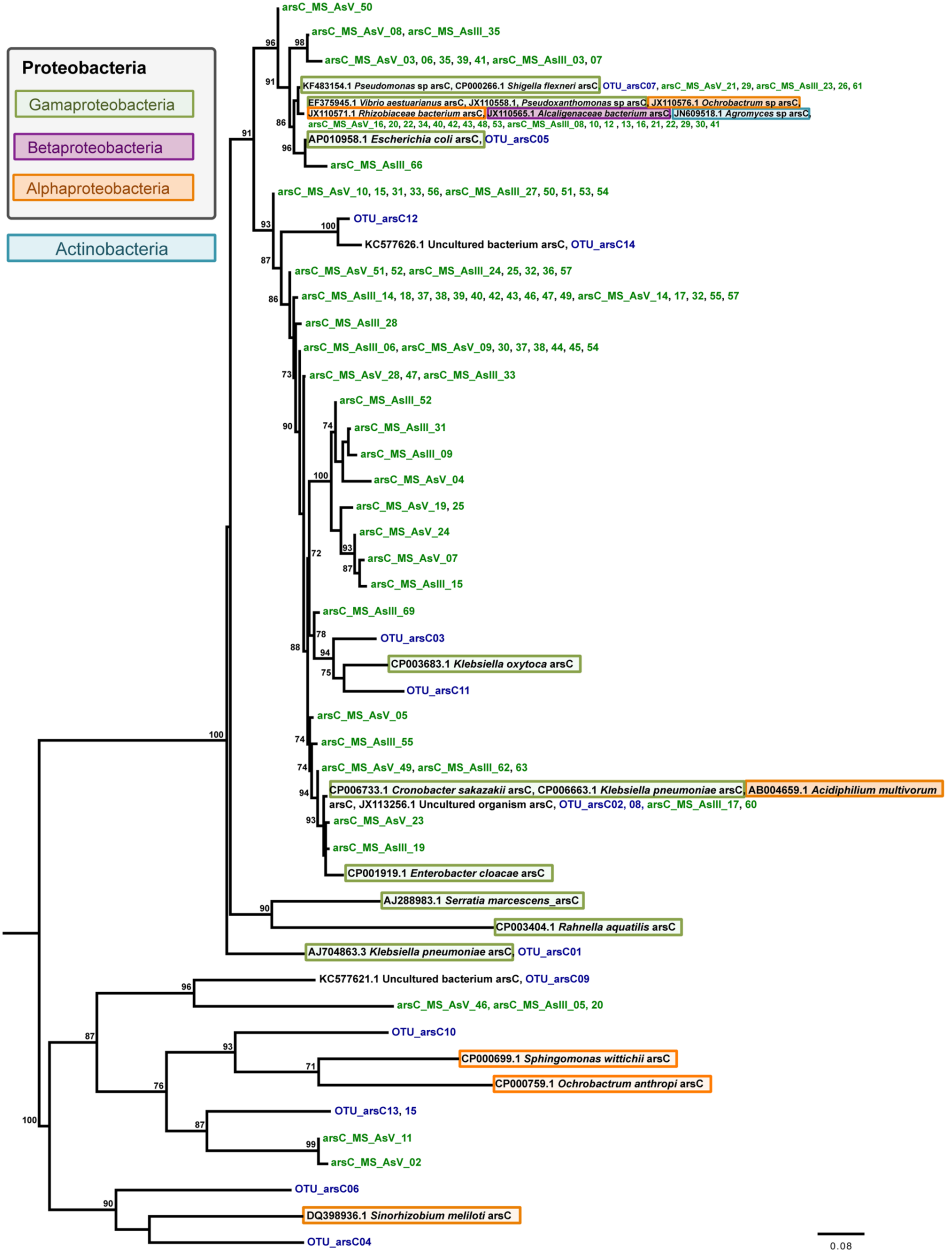
Evolutionary relationships of *arsC* sequences. A total of 48 nucleotide sequences and 352 sites were analyzed. The phylogeny was reconstructed by maximum likelihoodand TrN+I+G+F was selected as best fit model. Support values for each node were estimated using the Akaike Likelihood Ratio Test (aLRT). Only support values higher than 70% are shown. Reference sequences retrieved from the non-redundant database of the NCBI are shown in black, bacterial isolates (MS-AsIII and MS-AsV)in green, and operational taxonomic unities (OTUs) from As gene clone librariesin blue. Different background colors highlight *Actinobacteria* and three *Proteobacteria* classes – *Gamma-*, *Beta*, and *alpha-proteobacteria*.

**Figure 5:**
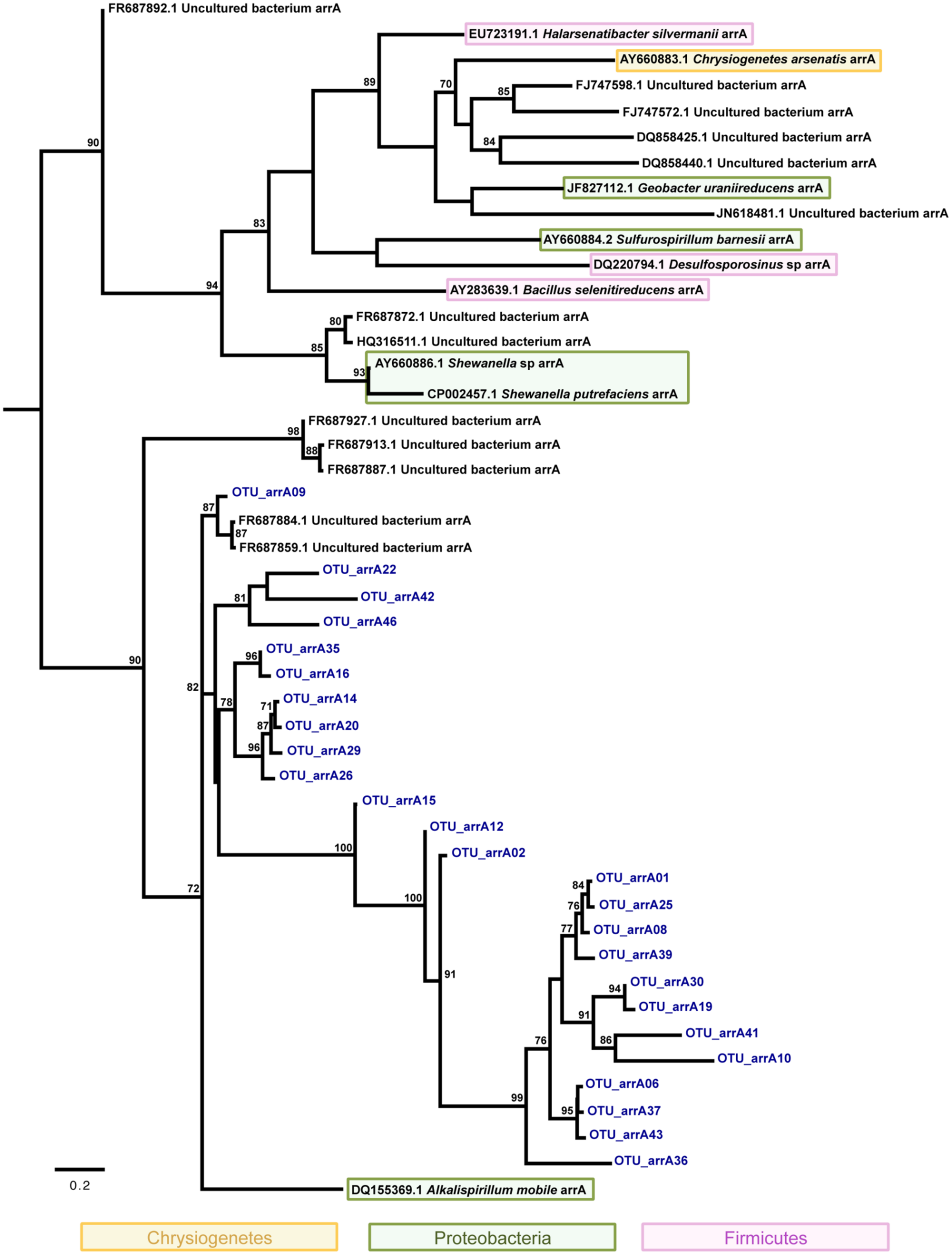
Evolutionary relationships of *arrA* sequences. A total of 47 nucleotide sequences and 242 sites were analyzed. The phylogeny was reconstructed by maximum likelihoodand GTR+I+G+F was selected as best fit model. Support values for each node were estimated using the Akaike Likelihood Ratio Test (aLRT). Only support values higher than 70% are shown. Reference sequences retrieved from the non-redundant database of the NCBI are shown in black, bacterial isolates (MS-AsIII and MS-AsV) in green, and operational taxonomic unities (OTUs) from As gene clone libraries in blue. Different background colors highlight three bacterial phyla - *Proteobacteria*, *Firmicutes*, and *Chrysiogenetes*.

**Figure 6:**
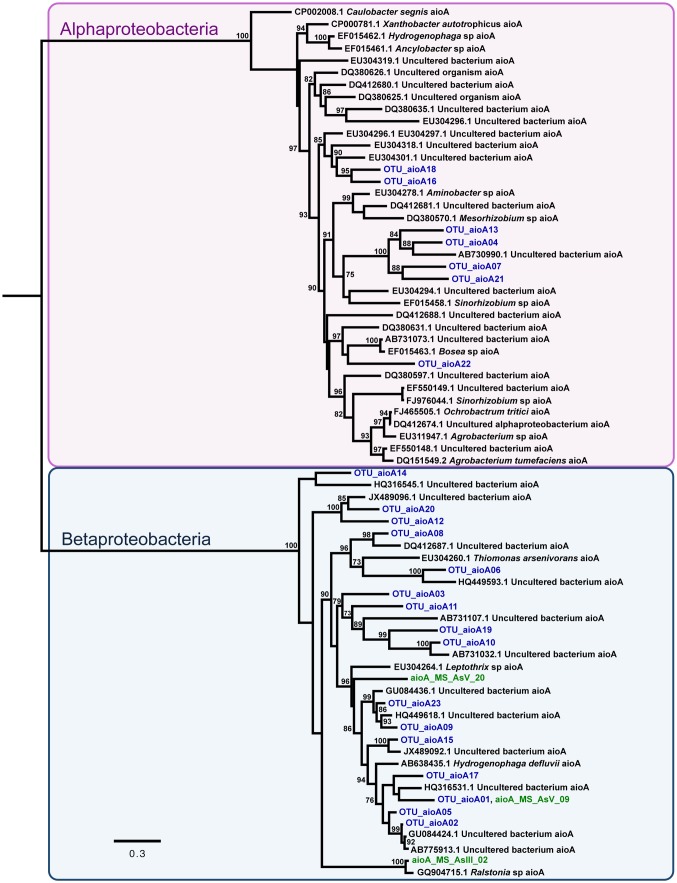
Evolutionary relationships of *aioA* sequences. A total of 72 nucleotide sequences and 543 sites were analyzed. The phylogeny was reconstructed by maximum likelihood and GTR+I+G+F was selected as best fit model. Support values for each node were estimated using the Akaike Likelihood Ratio Test (aLRT). Only support values higher than 70% are shown. Reference sequences retrieved from the non-redundant database of the NCBI are shown in black, bacterial isolates (MS-AsIII and MS-AsV) in green, and operational taxonomic unities (OTUs) from As gene clone libraries in blue. Different background colors highlight two Proteobacteria classes – *beta*- and *alpha-proteobacteria*.

Concerning evolutionary histories of As metabolism genes, the phylogenetic tree of *arsC* sequences was reconstructed with 48 nucleotide sequences and 352 sites, which represent 142 sequences (Fig. 4). TrN+I+G+F was selected as best fit model. The resulting phylogeny supports the hypothesis that horizontal gene transfer (HGT) seems to have played a role in the widespread distribution of *arsC* coding gene in *Actinobacteria* and *Proteobacteria*. Similar findings were retrieved on the phylogeny reconstructed for *arrA* sequences based on 47 nucleotide sequences and 242 sites where GTR+I+G+F was selected as best fit model (Fig. 5). On the other hand, the phylogenetic analysis of *aioA* sequences based on 72 nucleotide sequences and 543 sites shows two clades strongly supported: *alpha*- and *beta-proteobacteria* (Fig. 6) without clear evidence of HGT. For this analysis GTR+I+G+F was selected as best fit model. Interestingly, all putative *arrA* sequences obtained in this study (*arrA*- OTU) were more closely related to themselves or to sequences from uncultured bacteria, showing that more studies involving *arrA* sequences will be relevant to better understand the molecular diversity of those genes (Fig. 5).

## Discussion

The environmental impact of gold mining is presently a major concern because its processes release toxic metals such as As in both soil and groundwater. Considering the relevance of bacteria in the speciation of As in aquatic environments, we bioprospected As-resistant bacteria and As-transforming genes originated from sediments impacted by long-term gold mining. Although some studies have focused in the identification of As-resistant bacterial communities in a long-term As-contaminated environment [9]–[13], [49]–[52], the employment of combination of culture-based physiological and genomic approaches with metagenomic analysis in sediments collected from these areas are scarce [53], [54]. In this study, we reveal a large number of phylogenetically distinct As-resistant bacterial genera retrieved from sediment collected from a stream in a long-term gold-mining area.

We found *Bacillus* as the dominant genus in both MS-AsIII and MS-AsV enrichment cultures. Members of *Bacillus* are often found in As-contaminated environments [9], [11], [16], [54] being related to As-reduction and -oxidation, indicating that they are an essential component of As speciation in nature [54], [55]. The observed abundance of *Bacillus* isolates harboring the *arsC* and *aioA* genes confirmed its ubiquity and high As-resistance in As-rich environments, as it is the case of Mina stream sediment. This suggests an important role for *Bacillus* in As speciation. It also points to a possible use of these natural isolates in future bioremediation projects.

A recent study of our group [56], using culture-independent approach to assess the prokaryotic diversity in Mina stream sediment, revealed the presence of the *Thermomonas*, *Acidovorax*, *Acinetobacter* and *Ochobactrum* genera also detected in the present study. Moreover, Bandyopadhyay *et al*. [57] have proposed a novel species of the *Pannonibacter* genus, *Pannonibacterindica*, which is able to grow in high concentrations of AsV. However, it should be noted that *Thermomonas* and *Panonnibacter* were not previously reported in the literature as As-transforming genera.

The phenotypic and genotypic characterization of the MS-AsIII and MS-AsV bacterial isolates revealed their ability to reduce and oxidize As. Most bacteria (85%) were AsV-resistant bacteria (ARB) harboring the *arsC* gene, responsible for the reduction of AsV to AsIII, which is the most frequent detoxification reaction among bacteria in the environment [8]. Although the aerobic enrichment culture condition employed in this study could inhibit the growth of dissimilatory arsenate-reducing bacteria (DARB), it is likely that these bacteria were present because the *arrA* gene was detected. Several reports have evidenced DARB bioremediation potential of As-contaminated samples [2], [54], [58], [59].

AsIII-oxidizing bacterial isolates were minority (20%). This result is in agreement with those reported by Silver & Phung [14], who suggest that most isolates from natural environments lack AsIII-oxidizing ability. In this study, all AsIII-oxidizing isolates were classified as heterotrophic AsIII oxidizers (HAO) spanning 11 genera. However, only isolates belonging to *Bacillus*, *Pseudoxanthomonas*, *Stenotrophomonas*, *Micrococcus*, *Achromobacter*, and *Acidovorax* genera co-presented the oxidizing phenotype and genotype. From an ecological perspective, oxidizing bacteria are important performers in As-contaminated environments because they promote transformation from AsIII into AsV.

In a few isolates (8%), oxidizing and reducing As-transformation activities were not observed in their phenotype and genotype. There are several possible explanations for this. First, the As-transformation gene expression observed in isolates grown in the laboratory is likely to be different from that encountered in these isolates in nature, because of the different conditions of these environments. Second, these differences may reflect mutations in the As-resistance genes studied. Third, alternative resistance genes may be expressed by these isolates [60].

The high diversity and adaptability of the bacterial community disclosed herein could be explained by the presence of multiple copies of As-resistance genes either on bacterial chromosomes or on plasmids as a consequence of pressure created by the long-term contamination that occurs in the Mina stream area. Nevertheless, further studies will be needed to establish this.

Previous studies on As-resistance genes are associated with As-resistant cultivable isolates [10], [16], [59], [61]. Considering that the vast majority of bacteria are uncultivable, this traditional approach has limited our understanding of the extreme functional diversity in natural bacterial communities. Therefore, a metagenomic approach to investigate the functional genes associated with As-transformation in nature is essential to further our current knowledge on this matter. The analysis of *arrA* sequences revealed that all of them exhibited similarity with those from uncultured organisms. This predominance of uncultured organisms indicates that *arrA* gene present in Mina stream sediment is expressed by unidentified DARB. The *arsC* sequences detected in the sediment were similar to those previously reported [37], [62], [63]. The primers used in this study amplified *aioA*-like sequences [20], [64]. The *aioA* sequences were similar to several *aioA* genes of the *Proteobacteria* phylum. This finding is in agreement with Quéméneur *et al*. [65], who reported prevalence of AsIII-oxidizing *Proteobacteria* in mesophilic As-contaminated soils. However, it should be noted that *aioA* genes have been also detected in non-proteobacterial lineages [53], [69].

Phylogenetic analyses’ findings performed for MS-AsIII 16S rRNA, Ms-AsV 16S rRNA and As metabolism genes were consistent with findings obtained by similarity searches (blastx and blastn, respectively). Overall, the phylogenetic trees reconstructed for MS-AsIII and MS-AsV 16S rRNA sequences show very similar evolutionary histories where the relationships among *Firmicutes*, *Actinobacteria*, *Proteobacteria*, and *Thermodesulfobacteria* phyla members reflect the current knowledge regarding their evolution [66].

As previously described on the literature, the evolutionary relationships of *arsC* and *arrA* homologs (Figs.4 and 5) support the role of horizontal gene transfer (HGT) on the evolution of arsenate oxidases e.g. [67], [68]. The phylogeny reconstructed for *arsC* homologs (Fig. 4) clearly shows two *Ochrobactrum* sequences clustered in different well-supported clades suggesting that these two homologs were acquired by HGT from unrelated donors. Although it is known that due to HGT events *aioA* sequences are not a suitable marker for microbial diversity studies [53], it was not observed on the *aioA* phylogeny here presented (Fig. 6). Albeit *aioA* sequences have been detected in non-proteobacterial lineages [53], [69], our findings show two strongly supported clades clustering *alpha-* and *beta-proteobacteria* homologs. Such results probably reflect the bias existing on GenBank databases where most *aioA*sequences available are from proteobacterial lineages.

Overall, evolutionary analyses revealed high genetic similarity between some *arsC* and *aioA* sequences obtained from isolates and clone libraries, suggesting that those isolates may represent environmentally important bacteria acting in As speciation. In addition, some *arsC, aioA, and arrA* sequences were found to be closely related to homologs from uncultured bacteria. Thus, it may be hypothesized that these divergent sequences could represent novel variants of the As-resistance genes or other genes with related function. In addition, our findings show that the diversity of *arrA* genes is wider than earlier described, once none *arrA*-OTUs were affiliated with known reference strains. Therefore, the molecular diversity of arrA genes is far from being fully explored deserving further attention.

Altogether, this study is a bioprospection of AsIII-oxidizing and AsV-reducing bacteria and As-transforming genes in sediments impacted by long-term gold mining. Our culture efforts successfully identified a large number of phylogenetically distinct arsenic-resistant bacterial genera and revealed two novel As-transformation genera, *Thermomonas* and *Pannonibacter*. Our heterotrophic arsenite oxidizers and DARB isolates open new opportunities for their use in bioremediation of long-term gold-mining impacted areas. Furthermore, metagenomic analysis of As functional genes revealed a predominance of previously unidentified DARB.

## Supporting Information

Figure S1 **Map showing the sampling site. Crosshatch, red and yellow areas represent mining, urban, and sampling areas, respectively.**
(TIF)

Tables S1 **This file includes Table S1, S2 and S3.** Table S1. Phylogenetic affiliation of *aioA* OTUs based on blastx protein database. Table S2. Phylogenetic affiliation of *arsC* OTUs based on blastx protein database. Table S3. Phylogenetic affiliation of *arrA* OTUs based on blastx protein database.
(DOCX)
