# Supplementary figures and images for: Bacteria and Genes Involved in Arsenic Speciation in Sediment Impacted by Long-Term Gold Mining
Source: PLoS One. 2014 Apr 22;9(4):e95655. doi: 10.1371/journal.pone.0095655 (PMC3995719; doi:10.1371/journal.pone.0095655)

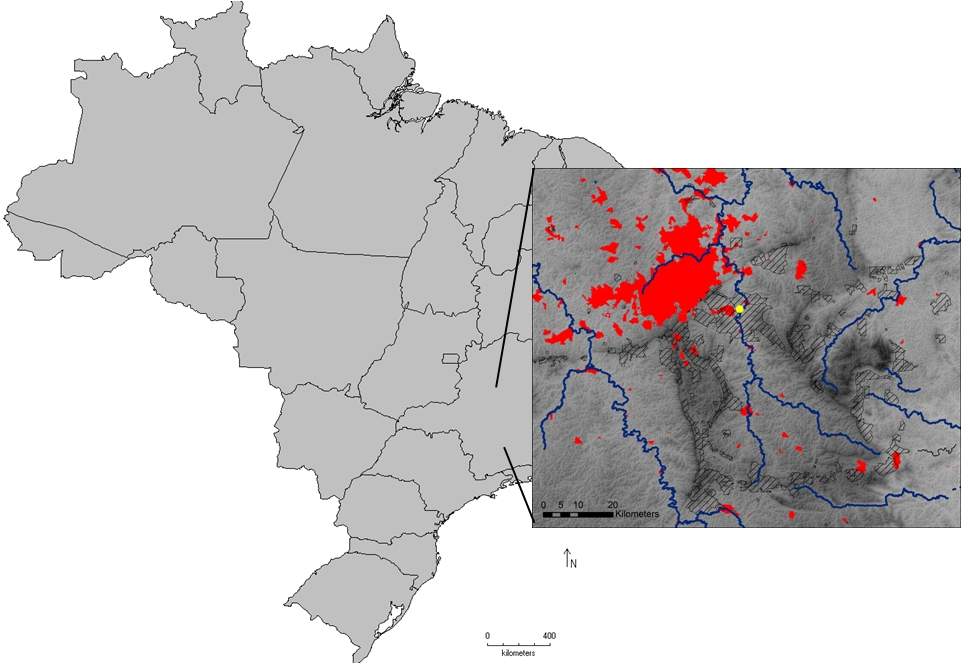

Supplement: Figure S1 — Map showing the sampling site. Crosshatch, red and yellow areas represent mining, urban, and sampling areas, respectively. (TIF) [file pone.0095655.s001.tif]
