# Supplementary material for: Bacteria and Genes Involved in Arsenic Speciation in Sediment Impacted by Long-Term Gold Mining
Source: PLoS One. 2014 Apr 22;9(4):e95655. doi: 10.1371/journal.pone.0095655 (PMC3995719; doi:10.1371/journal.pone.0095655)
Supplement: Tables S1 — This file includes Table S1, S2 and S3. Table S1. Phylogenetic affiliation of aioA OTUs based on blastx protein database. Table S2. Phylogenetic affiliation of arsC OTUs based on blastx protein database. Table S3. Phylogenetic affiliation of arrA OTUs based on blastx protein database. (DOCX) [file pone.0095655.s002.docx]

| OTU | Blastx Best Results | Organism (access number) | Id (%) | Source |
| --- | --- | --- | --- | --- |
| AIOA1 | arsenite oxidase large subunit | *Hydrogenophaga defluvii* (BAK39656.1) | 89 | Groundwater with arsenic |
| AIOA2 | arsenite oxidase large subunit | *Hydrogenophaga defluvii* (BAK39656.1) | 94 | Groundwater with arsenic |
| AIOA3 | arsenite oxidase large subunit | *Pseudomonas* sp. (AEL22192.1) | 78 | As-contaminated sediment from Sainte Marie aux Mines |
| AIOA4 | arsenite oxidase large subunit | uncultured bacterium (ADE33043.1) | 86 | arsenic contaminated water |
| AIOA5 | arsenite oxidase large subunit | *Hydrogenophaga defluvii* (BAK39656.1) | 94 | Groundwater with arsenic |
| AIOA6 | arsenite oxidase large subunit | uncultured bacterium (AEL22131.1) | 89 | As-contaminated sediment from Sainte Marie aux Mines |
| AIOA7 | arsenite oxidase large subunit | uncultured bacterium (AEC32846.1) | 92 | microbial mats from gold mine |
| AIOA8 | arsenite oxidase large subunit | *Thiomonas arsenivorans* (ABY19316.1) | 90 | As-contaminated soil from a disused gold mine |
| AIOA9 | arsenite oxidase large subunit | uncultured bacterium (AEL22137.1) | 93 | As-contaminated sediment from Sainte Marie aux Mines |
| AIOA10 | arsenite oxidase large subunit | uncultured bacterium (BAM24613.1) | 96 | aquatic sediment |
| AIOA11 | arsenite oxidase large subunit | *Thiomonas* sp. (ACA28597.1) | 71 | arsenic-rich acidic waters draining the Carnoules mine tailings |
| AIOA 12 | arsenite oxidase alpha subunit | *Burkholderia* sp. (ADF47196.1) | 77 | soil enrichment culture with arsenic |
| AIOA 13 | arsenite oxidase large subunit | uncultured bacterium (ADE33043.1) | 86 | arsenic contaminated water |
| AIOA 14 | arsenite oxidase alpha subunit | *Alcaligenes* sp. (ADF47197.1) | 76 | soil enrichment culture with arsenic |
| AIOA 15 | arsenite oxidase | uncultured bacterium (ABE02193.1) | 96 | arsenic contaminated sediment |
| AIOA 16 | arsenite oxidase large subunit | *Aminobacter* sp. (ABY19334.1) | 77 | As-contaminated mining site |
| AIOA 17 | arsenite oxidase large subunit | *Hydrogenophaga defluvii* (BAK39656.1) | 91 | Groundwater with arsenic |
| AIOA 18 | arsenite oxidase large subunit | *Aminobacter* sp. (ABY19334.1) | 80 | As-contaminated mining site |
| AIOA 19 | arsenite oxidase large subunit | uncultured bacterium (ABY19352.1) | 72 | As-contaminated soil |
| AIOA 20 | arsenite oxidase | uncultured bacterium (ABE02209.1) | 88 | arsenic contaminated sediment |
| AIOA 21 | arsenite oxidase large subunit | uncultured bacterium (AEC32846.1) | 84 | microbial mats from gold mine |
| AIOA 22 | arsenite oxidase alpha subunit | *Aminobacter* sp. (ABY19334.1) | 90 | As-contaminated mining site |
| AIOA 23 | arsenite oxidase large subunit | uncultured bacterium (ADE33054.1) | 96 | arsenic contaminated water |

Table S1 – Phylogenetic affiliation of *aioA* OTUs based on blastx protein database

Table S2 - Phylogenetic affiliation of *arsC* OTUs based on blastx protein database

| OTU | blastx best results | Organism (access number) | Id (%) | Source |
| --- | --- | --- | --- | --- |
| ARSC1 | arsenate reductase | *Klebsiella pneumoniae* (YP_002287000.1) | 100 | hospital |
| ARSC2 | arsenate reductase | *Cronobacter turicensis* (YP_003212785.1) | 100 | neonatal infections |
| ARSC3 | arsenate reductase | *Vibrio* sp. (ABO29820.1) | 92 | marine environments |
| ARSC4 | arsenate reductase | *Mesorhizobium alhagi* (ZP_09294313.1) | 82 | Symbiotic root nodules |
| ARSC5 | arsenate reductase | *Escherichia coli* (YP_001464969.1) | 100 | - |
| ARSC6 | arsenate reductase | *Methylocystis* sp. (ZP_08072529.1) | 76 | - |
| ARSC7 | arsenate reductase | *Vibrio* sp. (ABO29820.1) | 100 | marine environments |
| ARSC8 | arsenate reductase | *Cronobacter turicensis* (YP_003212785.1) | 100 | neonatal infections |
| ARSC9 | arsenate reductase | *Agrobacterium* sp. (ZP_08528114.1) | 95 | industrial strain |
| ARSC10 | arsenate reductase | *Novosphingobium* sp. (YP_004534179.1) | 92 | marine environments |
| ARSC11 | arsenate reductase | *Escherichia hermannii* (ZP_09808969.1) | 97 | - |
| ARSC12 | arsenate reductase | *Klebsiella oxytoca* (EHS95433.1) | 100 | - |
| ARSC13 | arsenate reductase | *Oligotropha carboxidovorans* (YP_002287046.1) | 83 | wastewater |
| ARSC14 | arsenate reductase | *Klebsiella oxytoca* (EHS95433.1) | 97 | - |
| ARSC15 | arsenate reductase | *Oligotropha carboxidovorans* (YP_002287046.1) | 83 | wastewater |

Table S3 – Phylogenetic affiliation of *arrA* OTUs based on blastx protein database

| OTU | blastx best results | Organism (access number) | Id (%) | Source |
| --- | --- | --- | --- | --- |
| ARRA1 | arsenate respiratory reductase | uncultured bacterium (CBW77469.1) | 83 | rock biofilms from an ancient gold mine |
| ARRA2 | arsenate respiratory reductase | uncultured bacterium (CBW77477.1) | 78 | rock biofilms from an ancient gold mine |
| ARRA6 | arsenate respiratory reductase | uncultured bacterium (CBW77469.1) | 83 | rock biofilms from an ancient gold mine |
| ARRA8 | arsenate respiratory reductase | uncultured bacterium (CBW77469.1) | 79 | rock biofilms from an ancient gold mine |
| ARRA9 | arsenate respiratory reductase | uncultured bacterium (CBW77469.1) | 98 | rock biofilms from an ancient gold mine |
| ARRA10 | arsenate respiratory reductase | uncultured bacterium (CBW77457.1) | 65 | rock biofilms from an ancient gold mine |
| ARRA12 | arsenate respiratory reductase | uncultured bacterium (CBW77477.1) | 75 | rock biofilms from an ancient gold mine |
| ARRA14 | arsenate respiratory reductase | uncultured bacterium (CBW77469.1) | 78 | rock biofilms from an ancient gold mine |
| ARRA15 | arsenate respiratory reductase | uncultured bacterium (CBW77477.1) | 74 | rock biofilms from an ancient gold mine |
| ARRA16 | arsenate respiratory reductase | uncultured bacterium (CBW77469.1) | 81 | rock biofilms from an ancient gold mine |
| ARRA19 | arsenate respiratory reductase | uncultured bacterium (CBW77469.1) | 83 | rock biofilms from an ancient gold mine |
| ARRA20 | arsenate respiratory reductase | uncultured bacterium (CBW77469.1) | 78 | rock biofilms from an ancient gold mine |
| ARRA22 | arsenate respiratory reductase | uncultured bacterium (CBW77469.1) | 78 | rock biofilms from an ancient gold mine |
| ARRA25 | arsenate respiratory reductase | uncultured bacterium (CBW77469.1) | 83 | rock biofilms from an ancient gold mine |
| ARRA26 | arsenate respiratory reductase | uncultured bacterium (CBW77469.1) | 81 | rock biofilms from an ancient gold mine |
| ARRA29 | arsenate respiratory reductase | uncultured bacterium (CBW77469.1) | 83 | rock biofilms from an ancient gold mine |
| ARRA30 | arsenate respiratory reductase | uncultured bacterium (CBW77469.1) | 84 | rock biofilms from an ancient gold mine |
| ARRA35 | arsenate respiratory reductase | uncultured bacterium (CBW77469.1) | 82 | rock biofilms from an ancient gold mine |
| ARRA36 | arsenate respiratory reductase | uncultured bacterium (CBW77469.1) | 78 | rock biofilms from an ancient gold mine |
| ARRA37 | arsenate respiratory reductase | uncultured bacterium (CBW77469.1) | 84 | rock biofilms from an ancient gold mine |
| ARRA39 | arsenate respiratory reductase | uncultured bacterium (AEX97846.1) | 64 | Cache Valley LandFill sediments |
| ARRA41 | arsenate respiratory reductase | uncultured bacterium (CBW77469.1) | 72 | rock biofilms from an ancient gold mine |
| ARRA42 | arsenate respiratory reductase | uncultured bacterium (CBW77469.1) | 69 | rock biofilms from an ancient gold mine |
| ARRA43 | arsenate respiratory reductase | uncultured bacterium (CBW77469.1) | 81 | rock biofilms from an ancient gold mine |
| ARRA46 | arsenate respiratory reductase | uncultured bacterium (CBW77477.1) | 72 | rock biofilms from an ancient gold mine |
